# N-Acetylcysteine and Allopurinol Synergistically Enhance Cardiac Adiponectin Content and Reduce Myocardial Reperfusion Injury in Diabetic Rats

**DOI:** 10.1371/journal.pone.0023967

**Published:** 2011-08-30

**Authors:** Tingting Wang, Shigang Qiao, Shaoqing Lei, Yanan Liu, Kwok F. J. Ng, Aimin Xu, Karen S. L. Lam, Michael G. Irwin, Zhengyuan Xia

**Affiliations:** 1 Department of Anaesthesiology, The University of Hong Kong, Hong Kong SAR, China; 2 Research Centre of Heart, Brain, Hormone and Healthy Aging, The University of Hong Kong, Hong Kong SAR, China; 3 Department of Pharmacology and Pharmacy, The University of Hong Kong, Hong Kong SAR, China; 4 Department of Medicine, The University of Hong Kong, Hong Kong SAR, China; University of Tor Vergata, Italy

## Abstract

**Background:**

Hyperglycemia-induced oxidative stress plays a central role in the development of diabetic myocardial complications. Adiponectin (APN), an adipokine with anti-diabetic and anti-ischemic effects, is decreased in diabetes. It is unknown whether or not antioxidant treatment with N-acetylcysteine (NAC) and/or allopurinol (ALP) can attenuate APN deficiency and myocardial ischemia reperfusion (MI/R) injury in the early stage of diabetes.

**Methodology/Principal Findings:**

Control or streptozotocin (STZ)-induced diabetic rats were either untreated (C, D) or treated with NAC (1.5 g/kg/day) or ALP (100 mg/kg/day) or their combination for four weeks starting one week after STZ injection. Plasma and cardiac biochemical parameters were measured after the completion of treatment, and the rats were subjected to MI/R by occluding the left anterior descending artery for 30 min followed by 2 h reperfusion. Plasma and cardiac APN levels were decreased in diabetic rats accompanied by decreased cardiac APN receptor 2 (AdipoR2), reduced phosphorylation of Akt, signal transducer and activator of transcription 3 (STAT3) and endothelial nitric oxide synthase (eNOS) but increased IL-6 and TNF-α (all *P*<0.05 vs. C). NAC but not ALP increased cardiac APN concentrations and AdipoR2 expression in diabetic rats. ALP enhanced the effects of NAC in restoring cardiac AdipoR2 and phosphorylation of Akt, STAT3 and eNOS in diabetic rats. Further, NAC and ALP, respectively, decreased postischemic myocardial infarct size and creatinine kinase-MB (CK-MB) release in diabetic rats, while their combination conferred synergistic protective effects. In addition, exposure of cultured rat cardiomyocytes to high glucose resulted in significant reduction of cardiomyocyte APN concentration and AdipoR2 protein expression. APN supplementation restored high glucose induced AdipoR2 reduction in cardiomyocytes.

**Conclusions/Significance:**

NAC and ALP synergistically restore myocardial APN and AdipoR2 mediated eNOS activation. This may represent the mechanism through which NAC and ALP combination greatly reduces MI/R injury in early diabetic rats.

## Introduction

Ischemic heart disease (IHD) is a major perioperative complication that is associated with significant morbidity and mortality, particularly in patients with diabetes [1]. Hyperglycemia-induced oxidative stress plays a central role in the development and progression of IHD in diabetes [2]. Mortality following myocardial infarction can be significantly reduced by procedures that allow the rapid return of blood flow to the ischemic zone of the myocardium, i.e., reperfusion therapy. However paradoxically, despite the restoration of coronary flow, reperfusion after a prolonged period of ischemia aggravates oxidative stress which is a major cause of myocardial injury. Recently, antioxidant therapy has become a promising pharmacological approach for the prevention of myocardial ischemia/reperfusion (MI/R) injury [3]. Although the rationale for the use of antioxidants is strong, antioxidant therapy with classic antioxidants in high-risk diabetic patients hasn't shown any beneficial effects on microvascular and macrovascular events [4]. Consequently, increasing attention has been paid to the development of interventions to decrease the risk of cardiovascular complications in diabetes.

In the myocardium, NADPH oxidase and xanthine oxidase are the two major enzymatic sources of reactive oxygen species (ROS) [5], [6]. N-acetylcysteine (NAC), the N-acetyl derivative of the amino acid cysteine, reduced NADPH oxidase activity [7]. However, NAC attenuated, but did not prevent myocardial dysfunction in diabetic rat hearts [7]. Allopurinol (ALP), a xanthine oxidase (XO) inhibitor, attenuated the diabetes-induced increased myocardial XO activity [8] and ROS production, and decreased oxidative/nitrosative stress, improving diabetes-induced cardiac dysfunction [9]. As NAC and ALP target different components of ROS, it is conceivable that NAC and ALP may synergistically reduce cardiac complications induced by oxidative stress, particularly in MI/R injury in diabetes.

Oxidative stress, even of short duration, can decrease the production of adiponectin (APN) [10], an adipocyte-derived plasma protein with anti-diabetic and anti-inflammatory properties [11], [12]. Plasma APN levels are decreased in patients with diabetes [13]. Deficiency in APN led to increased myocardial damage in response to ischemic insult and APN supplementation increased nitric oxide (NO) production and attenuates MI/R injury [14], [15]. APN can increase NO production by activating endothelial nitric oxide synthase (eNOS) [16]. However, in diabetes, myocardial eNOS protein expression is progressively reduced with increased nitrative and oxidative stress, while myocardial NO content is decreased [17]. In addition, studies have shown that APN may modulate oxidative stress and protect the heart from injury [18]. These investigations suggest that effective restoration of APN production by antioxidants could lead to attenuation or prevention of MI/R injury in diabetes.

Therefore, we hypothesized that NAC and ALP may synergize in reducing MI/R injury in diabetes, primarily through restoration of APN production or activation. We investigated the effects of antioxidants NAC and/or ALP on cardiac APN content and its receptors mediated signaling cascades and the effect on MI/R injury in the early stage of streptozotocin (STZ)-induced diabetic rats.

## Materials and Methods

### Induction of diabetes

Male Sprague Dawley rats (250±10 g, 6–8 weeks) obtained from the Laboratory Animal Service Center (University of Hong Kong) were used in this study. All rats were housed and given free access to standard rat chow and water in accordance with the principles of Animal Care of the University of Hong Kong. The experimental protocol used in this study was approved by the Committee on the Use of Live Animals in Teaching and Research (CULATR). Diabetes was induced by a single tail vein injection of STZ at the dose of 65 mg/kg bodyweight (Sigma-Aldrich, St. Louis, MO) in 0.1 M citrate buffer (pH 4.5) or citrate buffer alone as control under anesthesia with a combination of ketamine 67.7 mg/kg bodyweight and xylazine 6.77 mg/kg bodyweight. After 72 hours injection, blood glucose was measured using a One Touch Ultra Glucose meter (Life Scan Inc. USA) and rats with blood glucose levels over 15 mM were considered diabetic.

### Experimental protocol

Rats were randomly divided into five groups, control (C); diabetes (D); diabetes treated with NAC (Sigma-Aldrich, St. Louis, MO) (D+N); diabetes treated with ALP (Sigma-Aldrich, St. Louis, MO) (D+A) or their combination (D+N+A). The chemicals were dissolved in drinking water for 4-weeks duration of treatment starting one week after induction of diabetes. We adopted a dose of NAC at 1.5 g/kg/day, as our previous study has reported that NAC at the dose of 1.4–1.5 g/kg/day completely prevented hyperglycemia-induced oxidative stress after 8-week of STZ induction [19]. Also, the dose of ALP was adjusted to give a daily intake of 100 mg/kg/day in diabetic rats as described [20].

During the treatment period, general characteristics including water intake was assessed on a daily basis, while food consumption and body weight were monitored weekly. Plasma glucose levels (mM) were evaluated weekly using the One Touch glucometer (Life Scan Inc.USA). At termination (5-weeks after the onset of diabetes), rats were weighed and then euthanized with an intraperitoneal injection of pentobarbital sodium (65 mg/kg body weight). A blood sample was collected from the inferior vena cava, and plasma was extracted and stored at −80°C until further analysis. Hearts were removed from the chest and rinsed with ice-cold phosphate buffered saline, and weighed. The remaining ventricular tissue was immediately frozen in liquid nitrogen and stored at −80°C until assayed.

The subgroups of rats were subjected to MI/R by occluding the left anterior descending (LAD) artery for 30 min followed by 2 h reperfusion and myocardial infarction was determined as described below.

### Plasma and cardiac levels of free 15-F2t-isoprostane (15-F2t-IsoP)

Free 15-F2t-IsoP, a specific marker of oxidative stress in vivo originally produced by the random oxidation of tissue phospholipids, was measured by using an enzyme immunoassay kit (Cayman chemical, Ann Arbor, MI) as described [21]. Plasma samples and heart tissue homogenates (in PBS) were purified using Affinity Column and Affinity Sorbent (Cayman chemical, Ann Arbor, MI). The absorbance from the enzymatic reaction was detected at 412 nm. The values of plasma or cardiac free 15-F2t-IsoP were expressed as pg/mL in plasma or pg/g heart tissue in cardiac homogenates, respectively.

### Measurements of plasma and cardiac APN level

Plasma and cardiac APN levels were measured using a commercially available APN enzyme-linked immunosorbent assay (ELISA) kit (AdipoGen, Inc. Incheon, South Korea) following the manufacturer's protocol. In brief, frozen heart tissue or primarily cultured cardiomyocytes were homogenized at 4°C in cold buffer (20 mmol/L Tris-HCl, pH 7.5, 50 mmol/L 2-mercaptoethanol, 5 mmol/L EGTA, 2 mmol/L EDTA, 1 mmol/L PMSF, 10 mmol/L NaF, 25 µg/mL leupeptin, 2 µg/mL aprotinin) and then centrifuged at 1500 g for 5 minutes at 4°C. The supernatant was collected and stored at −80°C until analyses were conducted. The protein content of the samples was measured using the Bio-Rad protein assay kit with the use of bovine serum albumin as a standard. The values of plasma or cardiac APN were expressed as µg/mL in plasma or µg/mg in heart tissue or ng/mg in cardiomyocytes, respectively.

### Plasma biochemical analysis

Plasma TNF-α levels were determined using a rat TNF-α ELISA kit (eBiosource International, Burlington, Ontario, Canada). Plasma IL-6 levels were measured using a commercially available ELISA kit (eBiosource International, Burlington, Ontario, Canada). Plasma creatinine kinase-MB (CK-MB) levels were determined using a commercially available rat ELISA kit (R&D Systems, Minneapolis, MN).

### RNA extraction and RT-PCR

Total RNAs from heart tissue were extracted using TRIZOL obtained from Invitrogen (Carlsbad, Calif) following the protocol provided by the manufacturer. Extracted RNA was reverse transcribed to cDNA with the SuperScript™ II RNase H Reverse Transcriptase (Invitrogen, Carlsbad, Calif). Synthesized cDNA was amplified by Platinum TaqPCRx DNA Polymerase (Invitrogen, Carlsbad, Calif). Specific primers for APN: APN forward CTCCACCCAAGGAAACTTGT; APN reverse CTGGTCCACATTTTTTTCCT. PCR conditions for APN were 30 s at 94°C, 30 s at 55°C, and 40 s at 72°C for 30 cycles. The expression of a housekeeping gene, glyceraldehyde-3-phosphate dehydrogenase (GAPDH), served as internal control.

### Immunoprecipitation and western blot analysis

Frozen ventricular tissue samples (n = 6 rats/group) were homogenized in lysis buffer (20 mmol/L Tris-HCl pH = 7.5, 50 mmol/L 2-mercaptoethanol, 5 mmol/L EGTA, 2 mmol/L EDTA, 1% NP40, 0.1% SDS, 0.5% deoxycholic acid, 10 mmol/L NaF, 1 mmol/L PMSF, 25 mg/mL leupeptin, 2 mg/mL aprotinin), and protein concentrations were determined as previously described [22]. For direct immunoblotting, aliquots of lysate were mixed with 5×loading buffer containing 2-mercaptoethanol and maintained at 100°C for 10 min before loading on 10% SDS-PAGE. Following SDS-PAGE separation, proteins were transferred to PVDF membrane. Membranes were blocked in TBST containing 5% (w/v) non-fat milk and dried for 1 hour at room temperature [23]. Membrane strips were incubated with primary antibodies overnight at 4°C at the following dilutions: APN receptor 1 (AdipoR1) and APN receptor 2 (AdipoR2) (Santa Cruz Biotechnology, Santa Cruz, CA) 1∶500; total adenosine monophosphate–activated protein kinase-alpha (AMPK-α), Akt, signal transducer and activator of transcription 3 (STAT3), eNOS and GAPDH (Cell Signaling Technology, Beverly, MA) 1∶1000; phospho-AMPK-α, phospho-Akt (Ser 473), phospho-STAT3 (Ser 727) and phospho-eNOS (Ser 1177) (Cell Signaling Technology, Beverly, MA) 1∶500. Following extensive washing, membrane strips were incubated with a 1∶5000 anti-rabbit IgG (Cell Signaling Technology, Inc. MA) conjugated to horseradish peroxidase for 1 h. Protein bands were detected by a standard ECL method and images were measured by a densitometer with analysis software.

### Myocardial infarction and infarct size determination *in vivo*

After the completion of NAC and/or ALP treatments (5-weeks after the onset of diabetes), rats in the respective subgroups (n = 7/group) were anesthetized by intraperitoneal injection of sodium pentobarbital (65 mg/kg) and the trachea was cannulated with a polyethylene tube connected to a respirator with a tidal volume of 4 mL (60 breaths/min). Then left thoracotomy was performed between the fourth and fifth ribs. The pericardial tissue was removed under a microscope and the LAD artery was ligated with 8-0 silk suture using a snare occluder [24]. The rats were then subjected to 30 min of LAD ligation followed by 120 min of reperfusion. At the end of each experiment, myocardial infarct size was measured using TTC (1% 2,3,5-triphenyltetrazolium chloride) staining as described [25]. Briefly, the LAD was reoccluded and cannulated just distal to the occlusion site. Ten milliliters of saline and 10 mL of patent blue dye were injected at equal pressure into the LAD and left atrium, respectively, to delineate the anatomic area at risk (AAR) subjected to prolonged occlusion and reperfusion and the nonischemic normal zone. The heart was immediately fibrillated, removed, and sliced into serial transverse sections 6 to 7 mm in width. The unstained AAR was separated from the blue stained normal area, and the two regions were incubated at 37°C for 20 to 30 min in 1% TTC in 0.1 mol/L phosphate buffer adjusted to pH 7.4. TTC stains noninfarcted myocardium a brick red color because of the presence of a formazin precipitate, resulting from reduction of TTC by dehydrogenase enzymes present in viable tissue. Infarcted myocardium remains unstained. Infarcted and noninfarcted myocardium within the AAR were carefully separated and weighed after overnight storage in 10% formaldehyde. Infarct size (IS) was expressed as a percentage of the AAR.

Hemodynamics were continuously monitored using subcutaneous stainless-steel electrodes that were connected via a cable to a PowerLab monitoring system (ML750 PowerLab/4sp with MLT0380 Reusable BP Transducer; AD Instruments, CO Springs, CO).

### Rat cardiomyocytes isolation and cell treatment

Calcium-tolerant cardiac myocytes were prepared from adult male Sprague-Dawley rat ventricles using a modified method as described [26]. Briefly, rats were anesthetized and heparinized. The hearts were rapidly removed and mounted on a Langendorff perfusion apparatus, and immediately perfused with Ca^2+^-free buffer containing (in mmol/L) 120.4 NaCl, 14.7 KCl, 0.6 KH_2_PO_4_, 0.6 Na_2_HPO_4_, 1.2 MgSO_4_⋅7H_2_O, 10 Na-HEPES, 4.6 NaHCO_3_, 30 Taurine, 10 2,3-butanedione monoxime (BDM), 5.5 glucose (pH 7.1, 37°C). Enzymatic digestion was initiated by adding 1.5 mg/mL collagenase II (Invitrogen, Carlsbad, Calif) and CaCl_2_ (50 µM) to the perfusion solution. Following 20 min of digestion, the ventricle was quickly removed and gently teased into small pieces with fine forceps in the perfusion solution with 10% calf serum and 12.5 µM CaCl_2_. The solution was filtered with 70 µm nylon cell strainer (Fisher Scientific, Waltham, MA) to exclude un-digested tissues. After the cardiomyocytes were pelleted by gravity for 10 min, the supernatant was aspirated and the cardiomyocytes were resuspended with M199 medium with selenium/insulin/transferring (Sigma-Aldrich, St. Louis, MO) in 6-well polystyrene plates precoated with Matrigel. The final cell suspension, which contained about 70–80% viable myocytes, was subjected to the following experiments.

Recombinant adenoviruses for expression of either APN (Adv-APN) or control luciferase (Adv-Luc) was synthesized as described [27]. The cultured cardiomyocytes were infected with these adenoviruses at a multiplicity of infection of 50 as previously reported [28]. The cardiomyocyte preparations were randomly divided into four groups and treated as follows: (1) normal D-glucose (5.5 mM) medium as control group (C); (2) high D-glucose (25 mM) medium (H-G); (3) H-G group treated with Adv-APN (H-G+APN); (4) H-G group treated with Adv-Luc (H-G+Luc). After 48 h incubation, cells were collected and stored at −80°C until analyzed for APN concentration and AdipoR1 and AdipoR2 protein expression, respectively using ELISA and western blotting assays as described above.

### Statistical analysis

All the values are expressed as mean ± standard error of the mean (S.E.M.). One-way analysis of variance (ANOVA) was used for statistical analyses (GraphPad Prism, USA) of data obtained within the same group of rats and between groups of rats, respectively, followed by Tukey's test for multiple comparisons of group means. *P*<0.05 was considered statistically significant.

## Results

### General characteristics and plasma glucose levels

STZ-induced diabetic rats display hyperglycemia, polydipsia and polyphagia evidenced as significantly increased plasma glucose, water intake and food consumption, compared with age-matched controls (all *P*<0.05, Table 1). Treatment with NAC alone or NAC in combination with ALP markedly reduced food consumption and water intake (*P*<0.05), while ALP treatment alone slightly decreased water and food consumption. Both the body weight and heart weight in the diabetic group were lower than that in the control group (*P*<0.05), while the heart/body weight ratio in the diabetic group were higher than that in the control group (*P*<0.05). Although neither NAC alone nor the combination with ALP had a significant impact on body weight in diabetic rats, they prevented the increase in heart to body weight ratio seen in the diabetic group and normalized it to a level comparable to that in the control group (*P*<0.05, D+N or D+N+A vs. D). However, ALP alone had no effect on diabetic body weight and heart to body weight ratio. Plasma glucose was significantly elevated in the diabetic group as compared to that in the control. Neither NAC/ALP alone nor their combination had a significant impact on glucose level in diabetic rats.

**Table 1:** General characteristics after STZ treatment at termination of study.

Parameters	C	D	D+N	D+A	D+N+A
Water intake (ml/kg/day)	124.6±2.8	823.4±13.2*	400.5±7.2* #	690.6±10* #	453.1±7.2* #
Food consumption (g/kg/day)	68.5±4.7	179.1±13.3*	137.6±13.6* #	175.7±9.5*	158.8±9.06* #
Body weight (g)	489.7±15.3	314.9±9.5*	297.1±10.7*	295.6±15.6*	292.6±18.4*
Plasma glucose (mM)	6.2±0.3	28.4±1.1*	28.7±1.1*	27.8±1.1*	28.0±1.2*
Heart/Body weight ratio (g/kg)	3.5±0.2	4.3±0.3*	3.6±0.1#	4.1±0.2	3.7±0.2#

All values are expressed as Mean ± S.E.M. n = 7 per group. Water intake and food consumption were the average value of four weeks. Body weight, plasma glucose and Heart/Body weight ratio were measured at 4-week after STZ injection in these five groups.

*P<0.05 vs. C;

# *P*<0.05 vs. D.

### Oxidative stress marker free 15-F2t-IsoP levels

**Figure 1:**
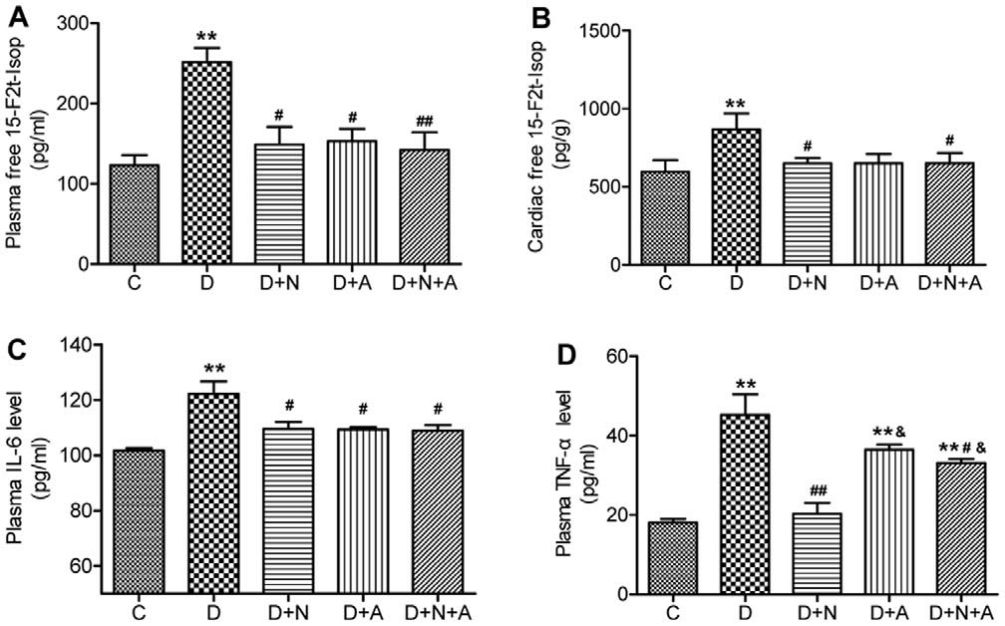
Plasma and cardiac tissue levels of 15-F2t-Isoprostane (15-F2t-Isop, A and B) and plasma levels of interleukin-6 (IL-6, C) and tumor necrosis factor-α (TNF-α, D) in control and diabetic rats with or without antioxidants treatment. Data are expressed as mean ± SEM (n = 7 per group), **P<0.01 vs. C group; ^#^ *P*<0.05, ^##^ *P*<0.01 vs. D group. ^&^ *P*<0.05 vs. D+N group.

As shown in Fig. 1A and 1B, both plasma and the cardiac tissue levels of free 15-F2t-IsoP were significantly elevated in diabetic rats compared to that of control rats (all *P*<0.01). NAC and ALP significantly reduced the level of free15-F2t-IsoP both in plasma and cardiac tissue (all *P*<0.05 vs. D). NAC in combination with ALP did not further reduce plasma and cardiac tissue free15-F2t-IsoP.

### Plasma IL-6 and TNF-α

Because APN is a protein with anti-inflammatory properties [29], we determined plasma IL-6 and TNF-α levels in control and diabetic rats with or without anti-oxidant treatment. As shown in Fig. 1C and 1D, plasma IL-6 and TNF-α levels were increased in the rats with diabetes. NAC and/or ALP treatment significantly reduced plasma IL-6 level in D rats, but had no synergistic effects. NAC significantly decreased plasma TNF-α level in D rats to a level comparable to that in the control group (*P*<0.01 D+N vs. D, *P*>0.05 D+N vs. C) while ALP had no significant effect on plasma TNF-α. Of note, NAC in combination with ALP evidently decreased plasma TNF-α in D rats (*P*<0.05 D+N+A vs. D) but the level remained significantly higher than that in the control (*P*<0.01 D+N+A vs. C, Fig. 1D).

### Cardiac and/or plasma APN expression/secretion

**Figure 2:**
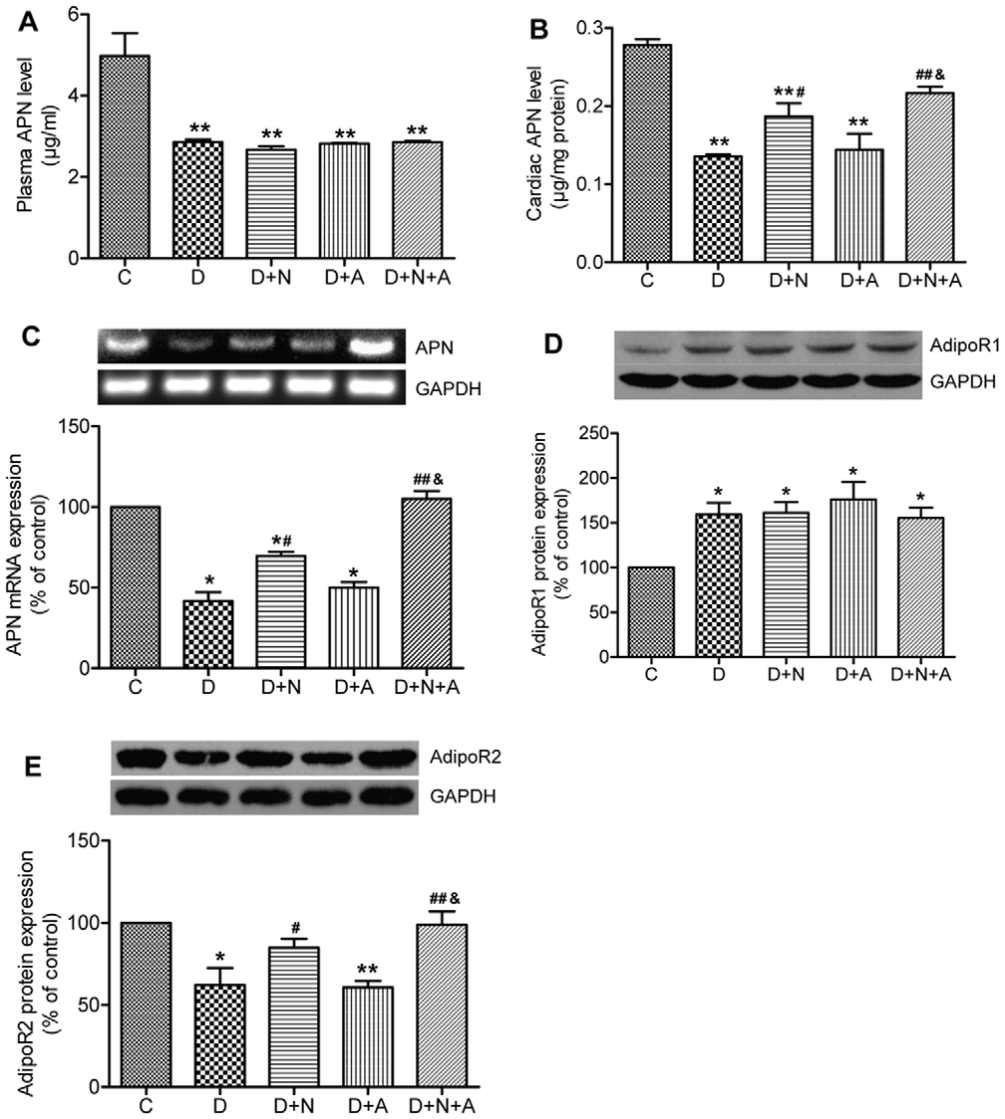
Plasma and cardiac adiponectin (APN) and myocardial APN receptors expression in control and diabetic rats with or without antioxidants treatment. A. Plasma APN levels. B. Cardiac APN levels. C. Cardiac APN mRNA expression. D. Cardiac AdipoR1 protein expression. E. Cardiac AdipoR2 protein expression. Data are expressed as mean ± SEM (n = 7 per group), *P<0.05, **p<0.01 vs. C group; ^#^ *P*<0.05, ^##^ *P*<0.01 vs. D group; ^&^ *P*<0.05 vs. D+N group.

To investigate whether the cardiac and/or plasma levels of APN are altered in rats in early stage of diabetes and whether or not they can be affected by antioxidants, we observed the effects of NAC and/or ALP on cardiac and plasma levels of APN in rats with STZ-induced diabetes. As shown in Fig. 2A and 2B, both plasma and cardiac APN levels at the 4^th^ week were decreased in D rats (*P*<0.01 vs. C). NAC partially but significantly restored cardiac APN concentration (*P*<0.05), but did not affect plasma APN level in D rats. ALP had no effect per se, but potentiated the beneficial effect of NAC on cardiac APN (*P*<0.05, D+N+A vs D+N). Changes in cardiac APN mRNA expression in various groups basically mirrored the changes in cardiac APN protein expression (Fig. 2C).

### APN receptors and the downstream signaling pathways

AdipoR1 and AdipoR2 serve as receptors for APN and mediate APN-related signaling pathways. We further investigated whether or not cardiac APN receptors and the downstream signaling proteins are altered in diabetic rats and whether they can be affected by antioxidants. As shown in Fig. 2D, the expression of cardiac AdipoR1 was increased in the early stage of rat diabetes compared with control rats, which was similar to the findings [30] observed in the late stage (8-weeks) of STZ-diabetic models. However, AdipoR2 expression was decreased in D rats (*P*<0.05 vs. C) (Fig. 2E). NAC but not ALP increased cardiac AdipoR2 expression (*P*<0.05), while neither of them affected cardiac AdipoR1 in D rats. ALP enhanced the effect of NAC in increasing cardiac AdipoR2 expression (*P*<0.05, D+N+A vs. D+N).

AMPK consists of one catalytic subunit (α) which plays an important role in cardiac metabolism and cardiomyocyte survival response to ischemia. Deficiency in APN resulted in AMPK-α inactivation at the late stage of STZ-diabetic rats [30]. However, it is unknown whether AMPK-α activation was altered in the early stage of diabetes and whether or not it could be affected by antioxidants. Therefore, we examined the cardiac AMPK-α protein expression and its phosphorylation status at Thr172 (p-AMPK-α, Thr172) as well as its downstream factor acetyl-CoA carboxylase (ACC). In contrast to its changes in the myocardium in the later stage of rat diabetes (15), cardiac phosphorylation of AMPK-α was significantly increased in the myocardium of early diabetic rats which was accompanied by increased ACC phosphorylation as compared with control rats (all *P*<0.05 vs. C). Activation of AMPK-α, as evidenced by increased phosphorylation at Thr-172, was attenuated by treatment with either NAC or ALP or their combination (all *P*<0.05, vs. D) although levels of p-AMPK-α at Thr-172 were still higher than that in the control. Of interest, neither NAC nor ALP alone had significant effects on enhanced ACC, while their combination significantly attenuated the increase in ACC phosphorylation seen in D rats.

**Figure 3:**
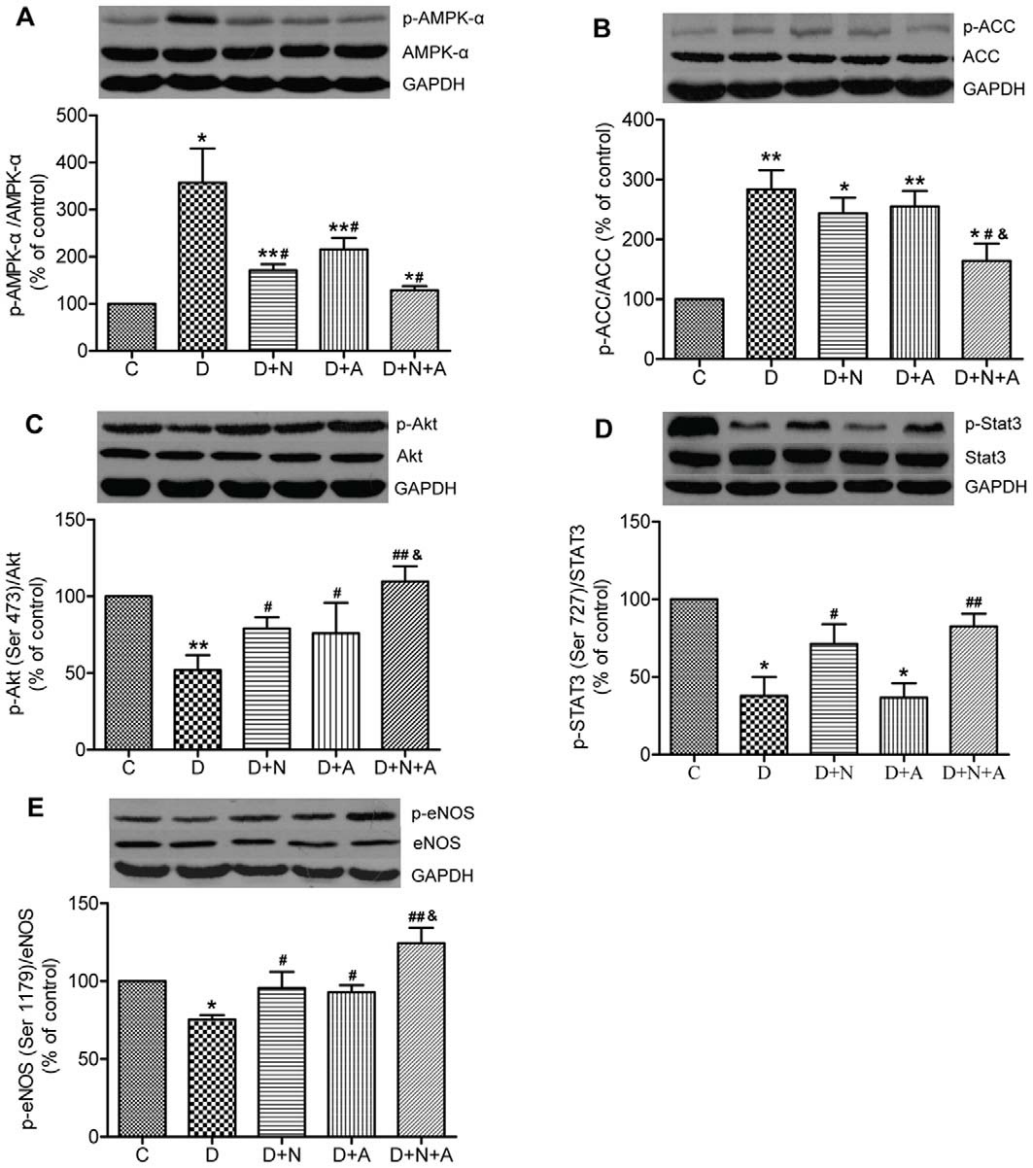
Cardiac protein expression of AMPK (A), ACC (B), Akt (C), STAT3 (D) and eNOS (E) and their phosphorylation status in control and diabetic rats with or without antioxidants treatment. Mean band density was normalized relative to GAPDH. Data are expressed as mean ± SEM (n = 7 per group), *P<0.05, **P<0.01 vs. C group; ^#^ *P*<0.05, ^##^ *p*<0.01 vs. D group; ^&^ *P*<0.05 vs. D+N group.

Studies show that phosphoinositide 3-kinase (PI3K)/Akt and janus kinase 2 (JAK2)/STAT3 signaling pathways may be involved in antioxidant-mediated NO production and subsequently contribute to myocardial protection from ischemia reperfusion. Furthermore, APN exerts its cardioprotection via eNOS mediated restoration of NO production. We, therefore, investigated changes in myocardial protein levels of Akt, STAT3 and eNOS as well as their phosphorylation status. As shown in Fig. 3C, 3D and 3E, myocardial phosphorylation of Akt, STAT3 and eNOS was significantly down-regulated in diabetic rats compared to control (*P*<0.05). Both NAC and ALP restored Akt and eNOS phosphorylation, while the combination conferred synergistic effects (*P*<0.05, D+N+A vs. D+N). NAC but not ALP restored STAT3 phosphorylation. STAT3 phosphorylation in the NAC and ALP combination group is about 1.2-fold of that in the D+N group, but the difference did not reach statistical significance.

### Hemodynamics and infarct size

To determine if antioxidants can attenuate MI/R injury in diabetes, we measured the hemodynamics and IS in the diabetic rats with or without antioxidants treatment. As shown in Table 2, baseline hemodynamic data were similar among groups, but a few significant (*P*<0.05) differences were observed (Table 2). Heart rate (HR) at baseline was not different among the five experimental groups. Coronary artery occlusion (ischemia) significantly decreased mean arterial pressure (MAP) and rate-pressure product (RPP) in all groups in comparison with baseline MAP. No significant differences in HR or RPP were observed between groups during ischemia and reperfusion. Combined NAC and ALP treatment facilitated recovery of MAP after reperfusion as compared to the diabetic untreated group.

**Table 2:** Hemodynamics at baseline, at 15 min of ischemia, at 1 h of reperfusion, and at 2 h of reperfusion in diabetic rats with or without antioxidants treatment.

	HR (bpm)	MAP (mmHg)	RPP (mmHg^.^min^−1^/1000)
Baseline			
C	275±12	125±4	34±1
D	303±13	117±3	36±2
D+N	308±16	117±6	36±1
D+A	318±9	117±8	38±3
D+N+A	309±10	117±4	37±2
Ischemia			
C	268±15	96±6**	26±3*
D	310±10	81±5** #	27±2*
D+N	304±18	82±2** #	27±2**
D+A	286±25	93±10	28±4
D+N+A	294±17	97±8**	28±2*
1 h reperfusion			
C	269±14	95±5**	25±1**
D	308±5	83±2** #	27±1**
D+N	309±18	83±4* #	29±3**
D+A	311±14	87±9*	29±3
D+N+A	310±18	90±8**	27±3*
2 h reperfusion			
C	290±22	90±7**	26±3*
D	290±7	75±3** #	24±1**
D+N	308±9	79±3** #	26±1**
D+A	309±13	84±9**	25±2*
D+N+A	300±14	85±6**	24±2**

All values are expressed as Mean ± S.E.M. n = 7 per group. HR, MAP and RPP were measured at baseline and during ischemia

* *P*<0.05,

** *P*<0.01 vs. their corresponding baseline;

# *P*<0.05 vs. their corresponding C groups.

**Figure 4:**
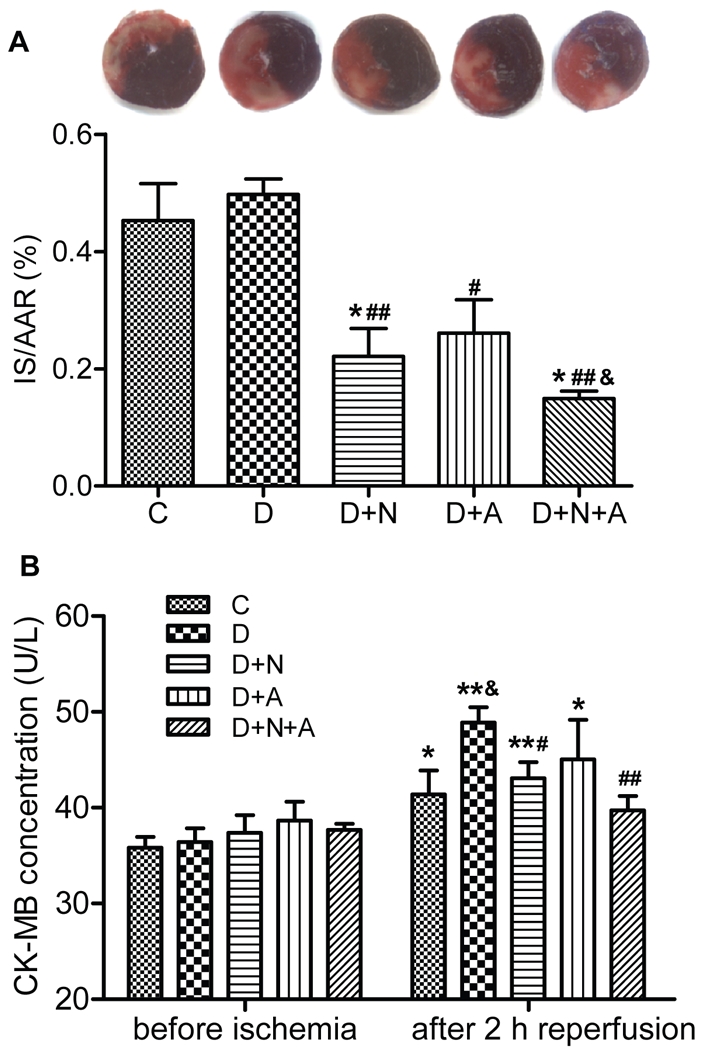
Myocardial ischemia reperfusion injury assessed after 30 min ischemia followed by 2 h reperfusion in control and diabetic rats with or without antioxidants treatment. A. Percent infarct size (IS) expressed as a percentage of the area at risk (AAR). Data are expressed as mean ± SEM (n = 7 per group). *p<0.05 vs. C group; ^#^ *p*<0.05, ^##^ *p*<0.01 vs. D group; ^&^ *P*<0.05 vs. D+N group. B. Plsma CK-MB secretion assessed by ELISA kit. Data are expressed as mean ± SEM (n = 7 per group). *p<0.05, **p<0.01 vs. C group before ischemia; ^&^ *p*<0.05 vs. C group after 2 h reperfusion; ^#^ *p*<0.05, ^##^ *p*<0.01 vs. D group after 2 h reperfusion.

The IS is shown in Fig. 4A. Coronary occlusion followed by reperfusion resulted in an IS of 45.3±14% of area at risk in control group and 49.7±5.9% in diabetic rats (*P*>0.05 D vs. C). NAC and ALP reduced IS to 22.1±9.9% (*P*<0.01) or 26.1±12.7%, respectively, as compared with the D group (all *P*<0.05 vs. D). The combination of NAC and ALP conferred a synergistic effect and further decreased IS to 14.9±3.0% (*P*<0.01 D+N+A vs. D, *P*<0.05 D+N+A vs. D+N or D+A).

### Plasma CK-MB levels

CK-MB isoenzyme is a major biomarker for myocardial cellular injury. As shown in Fig. 4B, there were no differences in CK-MB release among experimental groups before coronary occlusion. However, ischemia followed by reperfusion significantly increased plasma CK-MB concentration from 35.8±1.2 U/L to 41.4±2.5 U/L (*P*<0.05) in group C. Postischemic plasma CK-MB level after 2 hours reperfusion was significantly higher in D rats (*P*<0.05 D vs. C, Fig. 4B) which corresponded to a larger IS in D rats compared to C (Fig. 2A). NAC or ALP significantly reduced postischemic CK-MB release in D rats. The combination of NAC and ALP conferred a synergistic effect in attenuating postischemic CK-MB release in D rats to such a degree that by 2 h post-reperfusion, plasma CK-MB levels in the D+N+A group was similar to that before ischemia (*P*>0.05).

### Effects of cardiac APN on AdipoR2 expression in rat cardiomyocytes under high-glucose

**Figure 5:**
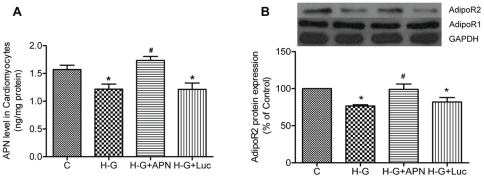
Cardiac adiponectin (APN) level affects cardiac APN receptors expression in rat myocardiocytes under high-glucose. A. Cardiomyocytes APN levels. B. Cardiomyocytes AdipoR1/2 protein expression. Data are expressed as mean ± SEM (n = 5 per group), *P<0.05 vs. C group; ^#^ *P*<0.05 vs. H-G group.

To determine whether or not cardiac APN level can affect the expression of APN receptors in diabetes, APN was supplied using an adenoviral transfection system into primary cultured rat cardiomyocytes under high glucose. As shown in Fig. 5A and 5B, both APN level and AdipoR2 protein expression in cardiomyocytes were significantly decreased after high glucose treatment for 48 h (*P*<0.05). Supplementation of APN to cardiomyocytes exposed to under high-glucose stimulation restored AdipoR2 protein expression to a level comparable to that in the control (*P*<0.05 H-G+Adv-APN vs. H-G; *P*>0.05, H-G+APN vs. C). However, cardiomyocyte AdipoR1 protein expression was not significantly altered after high-glucose treatment, nor was it affected by APN supplementation (Fig. 5B).

## Discussion

In this study, we have shown that both plasma and cardiac APN levels were decreased significantly in the early stage of type 1 diabetes in rats. NAC but not ALP increased cardiac APN concentration and AdipoR2 expression, while ALP enhanced the effects of NAC. To our knowledge, this is the first study to investigate the changes of cardiac APN level and its receptors in diabetic rats and the effectiveness of antioxidant treatment. Both NAC and ALP treatment decreased postischemic myocardial infarct size in the rats with diabetes respectively, while their combination conferred a synergistic effect in attenuating postischemic myocardial injury in diabetes. Further, we have shown that in cultured cardiomyocytes, high glucose exposure decreased cardiomyocyte AdipoR2 protein expression, which can be restored by APN supplementation.

It has been reported that oxidative stress negatively modulates APN gene expression [31], which means that APN could be decreased in diabetes as a result of hyperglycemia-induced oxidative stress. In STZ-induced type 1 diabetic rats, we found that both plasma and cardiac APN levels at the 4^th^ week were decreased compared with non-diabetic rats. Although some studies have shown that plasma APN levels are increased in type 1 diabetic patients, our study and other studies [30], [32] showed decreased plasma APN levels in type 1 diabetic rats and the reasons for this discrepancy may be due to species differences. As reported, constant high glucose significantly reduced APN protein expression and secretion compared to normal glucose conditions and this effect seems to be related to over-production of ROS, which can be attenuated by treatment with NAC [33]. Our study also showed that NAC restored cardiac APN concentrations, but did not affect plasma APN levels in rats with diabetes. Although ALP, similar to NAC, decreased oxidative stress biomarker free 15-F2t-Isop levels in plasma and cardiac tissue, ALP had no direct effect but potentiated the effect of NAC in enhancing cardiac APN levels. The reasons for this discrepancy between NAC and ALP remains unclear and merits further study. A possible explanation could be that NAC and ALP exert their antioxidant effect by targeting different initial activators which may have disparate effects in regulating APN expression. Of note, the inability of ALP to restore APN levels may explain why ALP treatment didn't prevent the development of myocardial hypertrophy in diabetic rats as evidenced by the increased heart-to-body weight ratio in the current study. It was reported that APN protects against the development of cardiac hypertrophy in diabetes [34]. Interestingly, despite of their similar antioxidant effectiveness in attenuating hyperglycemia-induced oxidative stress evidenced as reductions in plasma and cardiac levels of 15-F2t-IsoP, the effectiveness of NAC and ALP on heart weight and water intake varies. This may be potentially resulted from their different effects on secretion or sensitivity of APN, which can exert ameliorative effects on various symptoms of diabetes such as polydipsia and polyphagia via its anti-diabetic property. However, these hypotheses need to be further studied.

The proinflammatory cytokines TNF-α and IL-6 have been shown to both suppress and antagonize the action of APN in diabetes [35], [36]. In this study, both plasma TNF-α and IL-6 levels were significantly increased in rats with diabetes, which may contribute to the decreased plasma APN level in diabetes. Both NAC and ALP significantly decreased plasma IL-6 secretion in diabetes, but the combination conferred no further benefit. Of note, NAC alone but not ALP significantly decreased plasma TNF-α levels in group D. To be more precise, NAC treatment normalized plasma TNF-α to a level comparable to that in the control group. Notably, ALP partially attenuated the effect of NAC in decreasing plasma TNF-α levels, as such the plasma TNF-α concentration in the NAC and ALP combination group was significantly lower than that in group D but higher than that in NAC treated and in control group. This is an intriguing finding. The reason why ALP compromised NAC's effect in reducing TNF-α is not very clear. The ALP inhibition of xanthine oxidase activity should have altered the production of its metabolite uric acid, the later has been shown to react with NAC [37], which may potentially affect some of NAC's effects. Although plasma TNF-α concentrations were not completely consistent with plasma APN changes after antioxidant treatment in our study, the changes of plasma TNF-α seem to be inversely correlated to changes in cardiac APN levels. It is possible that, in diabetes, the cardiac origin of APN may play an important role in regulating systemic TNF-α. Study has shown that APN knockout mice exhibited high plasma TNF-α concentration [38] while supplementation of plasma APN decreased TNF-α in APN knockout mice, which supports the notion that APN can directly suppress TNF-α [39], [40]. These investigations by others and our current study demonstrated an anti-inflammatory effect of APN.

Studies have shown APN exerts its cardioprotective effect through AdipoR1 and AdipoR2, which are both expressed in the intact heart [41] and in cardiomyocytes [42]. AdipoR1 and AdipioR2 can be regulated under physiological and pathophysiological states, such as fasting [43], insulin [44], hyperglycemia [43], [45]. Current in vivo and in vitro studies showed that cardiac AdipoR2 protein expression was decreased under high glucose stimulation. However, cardiac AdipoR1 protein expression was increased in the in vivo model but not in cultured cardiomyocytes exposed to high glucose. The increased cardiac protein expression of AdipoR1 in diabetic rats may be a compensatory mechanism in response to decreased plasma and cardiac APN in the early stage of diabetes. NAC restored cardiac AdipoR2 expression, but did not affect cardiac AdipoR1 in diabetic rats. ALP had no effect itself, but potentiated the beneficial effect of NAC on cardiac AdipoR2 expression. Taken together, these findings suggest that enhancements of APN and cardiac AdipoR2 may represent the major mechanisms by which NAC or NAC and ALP combination confer cardioprotection in early diabetes. In addition, the good agreement between cardiac APN level and AdipoR2 expression in diabetic rats suggests that cardiac APN level may directly affects cardiac AdipoR2 expression under hyperglycemia. Indeed, our in vitro study demonstrated that high-glucose induced reduction of cardiomyocyte AdipoR2 protein expression can be restored by APN. The results suggest that cardiac APN may be a major contributor to the restoration of cardiac AdipoR2 expression in diabetes.

AMPK can be activated by APN via APN receptors [46]. However, our results showed that cardiac phosphorylation of AMPK-α was increased in diabetic rats at the early stage and this was accompanied by increased phosphorylation of downstream protein ACC despite reduction in cardiac APN and AdipoR2. It is possible that AdipoR1 activation plays a dominant role in APN mediated AMPK-α activation in early diabetes. It is also possible that diabetes-related hypoxia activated AMPK as reported [47]. NAC and/or ALP decreased phosphorylation of AMPK-α and ACC in diabetic rats, potentially as a result of attenuating oxidative stress or diabetic myocardial hypoxia. Although APN deficiency may lead to the inactivation of AMPK [48], which is contradictory to our finding, recent studies indeed suggest that the role of AMPK in cardiac function is controversial and AMPK activation may either be beneficial or detrimental to the ischemic heart [49]. The timing and magnitude of AMPK activation may determine the effectiveness of AMPK cardiac protection. In our study, antioxidant treatments attenuated the dramatic increase in AMPK activation, while maintaining its activation at a level moderately higher than that in the control group and led to beneficial cardioprotective effects. It has been well established that AMPK is the main kinase regulator of ACC, able to phosphorylate and inactivate ACC [50]. Excessive cardiac ACC phosphorylation by AMPK increases fatty acid oxidation which may exacerbate postischemic myocardial injury [51]. In our studies, NAC and ALP combination most prominently reduced both p-AMPK-α and p-ACC level in diabetic rats and resulted in most significant reduction of postischemic myocardial infarction in diabetes.

We have shown that APN can induce eNOS activation and increase NO production in human endothelial cells [28], prevent diabetic premature senescence of endothelial progenitor cells and promote endothelial repair [52]. The reperfusion injury signaling kinase (RISK) pathway including PI3K/Akt signaling cascade and the protective survivor activating factor enhancement (SAFE) pathway including JAK2/STAT3 signaling cascade are the most important pathways involved in eNOS activation and ischemia myocardial protection [53] [54]. Activation of Akt has been implicated in the control of skeletal muscle hypertrophy [55]. APN can also activate STAT3 in adult mouse cardiac fibroblasts [56]. Akt and JAK/STAT are congenerous pathways through which oxidative stress downregulates APN in 3T2-L1 preadipocyte cells [57]. Our studies showed that cardiac phosphorylation of Akt and STAT3 were decreased significantly in diabetic rats at the early stage followed by decreased eNOS activation. Both NAC and ALP significantly restored diabetic Akt and eNOS phosphorylation, while the combination conferred a synergistic effect. NAC alone significantly restored STAT3 phosphorylation. In contrast, ALP alone had no effect on STAT3 phosphorylation in diabetic rats, which was coincident with its inability to restore APN and AdipoR2 protein expression in diabetic rat hearts. Our results suggest that the antioxidants NAC and ALP may have conferred myocardial protection respectively primarily via the SAFE and/or the RISK pathways. Although cross-talk may exist between the RISK and SAFE pathways [54], they can work independently in mediating cardioprotection. Studies have reported that TNF-α, at moderately elevated concentrations, initiate the activation of SAFE pathway leading to cardioprotection [58], although at high concentrations TNF-α exert detrimental effects. In our study, ALP unexpectedly compromised the effect of NAC in reducing plasma TNF-α, such that the plasma TNF-α in the D+N+A group was lower than that in the diabetic group but yet higher than that in the non-diabetic control or NAC treated diabetic group, which resulted in the most prominent myocardial STAT3 phosphorylation and reduction of postischemic myocardial infarction in the D+N+A group. Although the underlying mechanism governing the synergy or interaction between NAC and ALP is yet to be investigated, our findings seem to be in agreement with the notion that moderate levels of TNF-α can stimulate the activation of SAFE pathway and thus confer cardioprotection [58].

Recently, it has been shown that low plasma APN is a clinical risk factor for myocardial infarction [59]. APN confers resistance to acute MI/R injury [14]. The restoration of cardiac APN levels after antioxidant treatment may contribute to the attenuation of postischemic myocardial infarction and cellular injury as well as hemodynamic improvement in diabetic rats because APN has beneficial actions on the cardiovascular system by directly acting on the heart and blood vessels [55]. Interestingly, a combination of NAC and ALP displayed a synergistic cardioprotective effect, which reinforces a strategy to use a combination of, rather than a single antioxidant, to inhibit ROS via different mechanisms. This study demonstrates for the first time the relationship between antioxidants, APN and MI/R injury. The results suggest that antioxidants modulate APN content, which may in turn enhance their cardioprotective effect.

In summary, this study demonstrated that, in the early stages of STZ-induced diabetes, rats displayed APN deficiency and increased inflammatory cytokines together with reduced cardiac AdipoR2 expression and impaired Akt-eNOS and STAT3-eNOS activation. The combination of NAC and ALP confer a synergistic effect on restoration of APN content and AdipoR2 mediated eNOS activation, which is a potential mechanism for enhancing the resistance to MI/R injury in the early stage of diabetes. Results from the present study provide insight into the effects and mechanisms of antioxidants synergy which may lead to the development of effective therapeutic regimens to combat diabetic myocardial complications.
